# Nurse practitioner consultations in primary health care: a case study-based survey of patients’ pre-consultation expectations, and post-consultation satisfaction and enablement

**DOI:** 10.1017/S1463423618000415

**Published:** 2018-07-17

**Authors:** Julian Barratt, Nicola Thomas

**Affiliations:** 1 Head of Community Nursing, Institute of Health, University of Wolverhampton, Wolverhampton, UK; 2 Professor of Kidney Care, School of Health and Social Care, London South Bank University, London, UK

**Keywords:** advanced clinical practice, communication, nurse practitioners, patient enablement, patient satisfaction

## Abstract

**Background:**

Research has not yet fully investigated links to consultation duration, patient expectations, satisfaction, and enablement in nurse practitioner consultations. This study was developed to address some of these research gaps in nurse practitioner consultations, particularly with a focus on expectations, satisfaction, and enablement.

**Aim:**

To explore the influence of pre-consultation expectations, and consultation time length durations on patient satisfaction and enablement in nurse practitioner consultations in primary health care.

**Design:**

Survey component of a larger convergent parallel mixed methods case study designed to conjointly investigate the communication processes, social interactions, and measured outcomes of nurse practitioner consultations. The survey element of the case study focusses on investigating patients’ pre-consultation expectations and post-consultation patient satisfaction and enablement.

**Methods:**

A questionnaire measuring pre-consultation expectations, and post-consultation satisfaction and enablement, completed by a convenience sample of 71 adults consulting with nurse practitioners at a general practice clinic. Initial fieldwork took place in September 2011 to November 2012, with subsequent follow-up fieldwork in October 2016.

**Results:**

Respondents were highly satisfied with their consultations and expressed significantly higher levels of enablement than have been seen in previous studies of enablement with other types of clinicians (*P*=0.003). A significant, small to moderate, positive correlation of 0.427 (*P*=0.005) between general satisfaction and enablement was noted. No significant correlation was seen between consultation time lengths and satisfaction or enablement.

**Conclusion:**

Higher levels of patient enablement and satisfaction are not necessarily determined by the time lengths of consultations, and how consultations are conducted may be more important than their time lengths for optimising patient satisfaction and enablement.

## Introduction

Patients’ evaluative perceptions of their clinical consultations have been analysed in research of communication in clinical consultations via three main areas of enquiry: expectations, satisfaction, and enablement. This paper focusses on a questionnaire-based analysis of patients’ pre-consultation expectations of consulting with nurse practitioners in primary care, and subsequent post-consultation patient satisfaction and patient enablement.

The term patients’ expectations are linked to that of patient satisfaction, as evaluative satisfaction with health care is often dependent on the type of care a patient expected to receive, which would imply that expectations of care must be assessed before measuring satisfaction. Comprehending the formation of patient expectations and their subsequent effects on consultation interactions and outcomes has been noted as important to ensure a complete understanding of patient–clinician consultations (Stevenson *et al*., 2000; Ford *et al*., 2003; Redsell *et al*., 2007a; Pawlikowska *et al*., 2009).

Patient satisfaction is a multi-dimensional and dynamic process, involving judgement of the interrelated physical, psychological and social elements of a consultation, which does not always have to end in the production of a list of dissatisfied or satisfied features of health care service provision, but instead can also strive to analyse and understand patients’ experiences of health care (Green and Davis, 2005; Thrasher and Purc-Stephenson, 2008).

Partly in response to critiques of the diverse, multi-dimensional nature of patient satisfaction, the more specific concept of patient enablement has been developed in consultation communication research (Desborough *et al*., 2017a; 2017b). Patient enablement goes further than the concept of patient satisfaction as it moves beyond the consultation to consider whether patients feel more able to manage their health as a result of consulting with a clinician, rather than solely focussing on an evaluation of the care provided by their clinician, as measures of patient satisfaction typically do (Collins *et al*., 2007; Frost *et al*., 2017). The conceptual basis of patient enablement is that post-consultation patient outcomes such as satisfaction are determined by how patients feel after their consultations; the premise being that patients who do feel more enabled will exhibit higher levels of satisfaction (Andén *et al*., 2006).

## Background

Patients sometimes have uncertain expectations of consulting with nurses working in advanced clinical roles, such as nurse practitioners, for example, thinking that seeing the nurse is just an interim care measure and that they would then still need to see a medical doctor for receiving definitive care (Redsell *et al*., 2007a). It has also been speculated that patients’ lowered expectations of consulting with nurse practitioners may affect patients’ subsequent evaluations of consultations via outcome measures, such as satisfaction, though this relationship has not yet been fully examined (Horrocks *et al*., 2002; Redsell *et al*., 2007b). Accordingly, it is appropriate to further examine patients’ expectations and evaluative perceptions of consulting with nurse practitioners, and also to determine the relationship between patients’ expectations, satisfaction, and enablement.

It has frequently been noted in North American studies of nurse practitioner consultations that many patients report high levels of satisfaction after consulting with a nurse practitioner (Knudston, 2000: Pinkerton and Bush, 2000; Agosta, 2009a; 2009b). However, in the United Kingdom, whilst high levels of patient satisfaction with nurse practitioner consultations have also been recorded, they are not always consistently found in UK-based studies (Kinnersley *et al*., 2000; Horrocks *et al*., 2002). A point of difference is that in North American studies of patient satisfaction with nurse practitioner primary health care consultations, satisfaction has often been measured using specially designed instruments for measuring patient satisfaction with nurse practitioner consultations (Knudston, 2000; Agosta, 2009a; 2009b), whilst in the United Kingdom in currently available larger studies of patient satisfaction with nurse practitioner consultations in primary health care, satisfaction has typically been measured with instruments originally developed to measure patient satisfaction with medical doctor consultations (Kinnersley *et al*., 2000; Venning *et al*., 2000; Horrocks *et al*., 2002). Consequently, it is important to also investigate patient satisfaction with nurse practitioner primary health care consultations in the United Kingdom with an instrument specifically devised for measuring satisfaction in those types of consultations.

In comparison to what is already known about patient satisfaction with nurse practitioners, little is known about how enabled patients are to manage their health after consulting with a nurse practitioner, as there has been only minimal investigation of this phenomenon focussed on nurse practitioners (Charlton *et al*., 2008), though patient enablement has recently been investigated in relation to practice nurses by Desborough *et al*., 2017a, though the nurses in that study were not practising at an advanced level of practice. Therefore, it is appropriate to further investigate how enabled patients feel after consulting with a nurse working at an advanced level of practice, such as nurse practitioners. Furthermore, there has been a minimal investigation of the potential associative relationship between patient satisfaction and patient enablement after consulting with nurse practitioners, so that relationship also requires analysis (Barratt, 2016).

A further factor to consider in relation to patients’ evaluative perceptions of nurse practitioner consultations is the time length of those consultations. In some studies of nurse practitioner consultations patients have often qualitatively reported the sense of having more time to speak with nurse practitioners in consultations than they do with medical doctors (Barnes *et al*., 2004; Williams and Jones, 2006), and nurse practitioners have also qualitatively recounted a similar sense of having more time to consult with patients (Kleiman, 2004). Quantifiably, in currently available systematic reviews of the outcomes of nurse practitioner consultations, the mean time lengths of nurse practitioner consultations are significantly longer than those of medical doctor consultations (Horrocks *et al*., 2002; Laurant *et al*., 2005). Such findings have led some researchers to speculate that the increased time lengths of nurse practitioner consultations and the resultant space they allow for additional social interactions to occur, may explain the higher levels of patient satisfaction often reported for nurse practitioner consultations (Seale *et al*., 2005; 2006), though that relationship has not yet been adequately explored in research of nurse practitioner consultations. It is important to determine the time length of contemporary nurse practitioner consultations, as the prior systematic reviews of the outcomes of nurse practitioner consultations were conducted some time ago before the widespread expansion of advanced nursing practice and nurse independent prescribing in the United Kingdom (Horrocks *et al*., 2002; Laurant *et al*., 2005; Bonsall and Cheater, 2008). Furthermore, as it has also not yet been objectively determined if there is a relationship between the independent variable of nurse practitioner consultation time length and the dependent variables of either patient satisfaction or patient enablement, it is also apt to examine those consultation time length relationships.

## Study design, aim, and research questions

This paper presents the findings of the survey component of a larger convergent parallel mixed methods case study of communication in nurse practitioner consultations (Barratt, 2016; 2018). The mixed methods case study was designed to conjointly investigate the communication processes, social interactions, and measured outcomes of nurse practitioner consultations. The other components of case study data collection comprised video recordings of nurse practitioner consultations, and semi-structured interviews with the patient, carer, and nurse practitioner participants of the video recorded consultations; the findings of those other components of the mixed methods study are reported elsewhere in *Primary Health Care Research & Development*.

Creswell (2014: 2) defines mixed methods research as: ‘an approach to research … in which the investigator gathers both quantitative (closed-ended) and qualitative (open-ended) data, integrates the two, and then draws interpretations based on the combined strengths of both sets of data to understand research problems’. This definition has been applied in this study as a consensually representative opinion of mixed methods research, which in turn guided the developmental mixed methods design of the overall study. A convergent parallel mixed methods design involves the separate collection and analysis of both quantitative and qualitative data, followed by comparative merging and interpretation of the data sets in a succeeding discussion (Creswell, 2013; 2014). In this study, a convergent parallel mixed methods design was selected so as to enable concurrent collection of quantitative and qualitative data during field visits, thus making an expedient use of the time available for data collection. A convergent parallel design also enables a researcher ‘to gain multiple pictures of a problem from several angles’ (Creswell, 2014: 37), which in this case study is the nurse practitioner consultation, and therefore also supports the convergence of data collection upon the phenomenon being studied. Creswell and Plano Clark (2010: 73) note the overall purpose of a convergent parallel design is to facilitate a more ‘complete understanding of a topic’ and in doing so equal emphasis is normally placed on the priority of qualitative and quantitative strands within a convergent mixed methods design.

The case study setting was a primary health care clinic in an urban area of England providing general practice services, where the majority of registered patients consult with nurse practitioners for both same day and pre-booked appointments for the assessment and management of both acute medical problems and long-term conditions.

The aim of this survey component of the study was to explore the influence of patient pre-consultation expectations, and consultation time length durations on patient satisfaction and patient enablement in nurse practitioner consultations.

The research questions addressed in the survey component of the case study were:

- What are patients’ expectations of consulting with nurse practitioners?
- Do patients’ expectations of consulting with nurse practitioners affect their subsequent evaluations of post-consultation satisfaction and enablement?
- From a UK perspective how satisfied are patients after consulting with nurse practitioners when satisfaction is measured with an instrument specifically devised for measuring satisfaction with those types of consultations?
- How enabled are patients to manage their own health after consulting with a nurse practitioner?
- Do the outcome variables of patient satisfaction and patient enablement after consulting with nurse practitioners have any associative relationship?
- Does the time length duration of nurse practitioner consultations affect the outcomes of patient satisfaction and enablement?

## Methods

Satisfaction and enablement data was collected in the survey component of the case study using two previously validated questionnaires: the ‘Nurse Practitioner Satisfaction Survey’ (NPSS), which has been specifically developed in North America for measuring patient satisfaction with nurse practitioner delivered primary care (Agosta, 2009a); and a frequently used measure of patient enablement, developed in the United Kingdom, called the ‘Patient Enablement Instrument’ (PEI), which is intended to capture patients’ feelings of confidence, ability and, coping after a general practice consultation (McKinley, 2004). Additionally to measure patients’ expectations of the nurse practitioner consultation, activities that are typically undertaken in medical general practice consultations such as history taking, diagnosis, prescribing, and referrals were identified to develop items measuring patients’ probability expectations of what they thought would actually happen in relation to their prospective consultation. An additional questionnaire item in the expectations section asked if respondents expected the nurse practitioner to discuss their case or that of the person they were accompanying with a doctor. This extra item was designed to assess whether or not respondents fully understood the autonomous nature of the nurse practitioner role, as nurse practitioners do not routinely need to discuss the patients they see with a doctor.

### Participants

In the video recording component of the case study, a convenience sample of 30 people registered at the selected clinic, consulting with three nurse practitioners employed at the selected clinic was recruited, and those 30 participants were also asked to complete a questionnaire. Additionally, to diversify the survey sample, a further convenience sample of 70 people whose consultations at the selected clinic had not been video recorded were also asked to complete a questionnaire. The purpose of asking a group of participants whose consultations were not video recorded to complete the questionnaire was first to allow comparison with the video recorded participants to check that satisfaction and enablement was not affected by the consultation observation; and second to get a better measure of patient satisfaction and enablement arising from nurse practitioner consultations. All participating patients were attending for either same day or pre-booked appointments.

### Data collection

A pilot study for the questionnaire used in the study was conducted in June 2011. The research case study’s initial fieldwork took place over a 14-month period starting in September 2011 and finishing in November 2012. This first fieldwork period comprised nine field visits, totalling ~35 h divided over the nine visits. The ensuing detailed data analysis for the case study was completed between 2012 and 2016. A second follow-up episode of fieldwork was completed in October 2016, involving presenting the findings to the nurse practitioner participants at the selected clinic, to enable them to have a respondent validation opportunity to challenge, discuss, and reflect as a group on the case study’s findings arising from the videoed consultations, survey, and interviews to facilitate engagement with, and add to, the previously analysed data (Birt *et al*., 2016) The respondent validation comments were then applied to the case study’s findings to additionally reflect the nurse practitioner participants’ interpretations of the video, survey, and interview data.

### Data analysis

The questionnaire data were inputted and analysed using IBM SPSS Statistics 20. All statistical tests were conducted as two-tailed with significance measured at the 0.05 level. Non-parametric tests were mostly, though not exclusively, selected for exploratory analysis, as the sample sizes in the study were relatively small, and the skewness statistics for most of the data indicated it was not normally distributed (Gliner *et al*., 2017). An exception to this was the data for enablement which where the skewness statistic were calculated as under 1, indicating it was more normally distributed. Therefore, parametric tests were used for exploratory analysis of the enablement data.

Descriptive statistics were used to summarise the demographic profiles of the questionnaire respondents and to summarily describe respondents’ pre-expectations of the nurse practitioner consultation. One-sample Binomial tests were used to determine any significant differences in pre-consultation expectations amongst demographically defined groups of respondents. The sample mean and median satisfaction and enablement scores were calculated. Once the overall satisfaction scores had been determined Mann–Whitney *U* tests were used to investigate if there were any significant differences in respondents’ satisfaction scores variability in relation to binary variables such as being video recorded versus not being video recorded; gender; and ethnicity. Kruskall–Wallis *H* tests were used to determine if there were any significant differences in respondents’ satisfaction scores in relation to categorical variables with more than two categories such as age, and the different nurse practitioners seen. Once the overall enablement scores had been ascertained independent samples *t*-tests were used to find out if there were any significant differences in respondents’ enablement scores in relation to binary variables such as being video recorded versus not being video recorded; gender; and ethnicity. Analysis of variance *F* tests were then used to discover if there were any significant differences in respondents’ enablement scores in relation to categorical variables with more than two categories such as age, and the different nurse practitioners seen. The respondents’ satisfaction scores were compared with their pre-consultation expectations using Mann–Whitney *U* tests. Independent samples *t*-tests were used to determine if there were any significant differences in respondents’ enablement scores variability in relation to their pre-consultation expectations. A correlational analysis of the satisfaction and enablement scores was performed, using Spearman’s *ρ*, to ascertain if any associative relationship existed between the two variables. The video recorded consultation time lengths were also correlated using Spearman’s *ρ*, with the scores for satisfaction and enablement, to see if there was any relationship between consultation time lengths and those variables.

### Validity, reliability, and rigour

Permission was sought to use the NPSS from its creator Agosta (2009a). The NPSS has a high Cronbach’s *α* reliability coefficient of 0.98 (Agosta, 2009b). The PEI also has a high Cronbach’s *α* reliability coefficient of 0.92 (Howie *et al*., 1998). Prior permission was not sought to use the PEI in the study as the instrument is freely and publically available from multiple websites and other published surveys of patient enablement.

The questionnaire comprised 51 items divided over four discrete sections: pre-consultation expectations; post-consultation satisfaction; post-consultation enablement; and demographic information. The satisfaction section enables determination of two Likert-scale measurements of patient satisfaction: general satisfaction (maximum possible score 85) and communication satisfaction (maximum possible score 30). The PEI-derived section comprises six items with a possible score of 0–12, with a higher score indicating more enablement (Wensing *et al*., 2007).

Before the main study data collection started the questionnaire was piloted with five general practice patients and five clinical academic nurse practitioners to examine its perceived functionality. All of the pilot study participants found the questionnaire easy to complete and suggested only minor formatting changes, which were incorporated in the final version of the questionnaire.

## Results

### Questionnaire responses

Questionnaire responses were provided by a convenience sample of 71 adult respondents in a general practice clinic, including 26 respondents whose consultations had been video recorded. 100 hard copy questionnaires were made available for distribution at the clinic. In total, 30 questionnaires were designated for use with the video recorded participants, of which 26 were completed, and the remaining 70 questionnaires were placed at reception, and the receptionists were asked to give the questionnaires to any patients attending for nurse practitioner appointments which were not being video recorded, of which 45 were completed. The combined response rate for the questionnaires was 75.4%.

### Demographic details of respondents

In overview the majority of questionnaire respondents reported their gender as female (*n*=48, 71.6%); were aged 36-65 years old (*n*=38, 53.5%); and were either married or living with their partner (*n*=40, 62.5%). In relation to highest education level completed the majority of respondents were educated to university degree level (*n*=38, 61.3%). A large majority of respondents described themselves as White (*n*=51, 75%). The majority of respondents placed themselves in the £10 000–£40 000 income bracket (*n*=26, 53.1%). Over half of the respondents described themselves as being employed (*n*=39, 58.2%).

### Pre-consultation expectations

For the patients’ expectations data a one-sample Binomial test was used to determine if there was any significant difference in the proportions responding ‘Yes’ and ‘No’ for the different pre-consultation expectations of activities participants were expecting to see in their consultations. The results of this analysis of pre-consultation expectations are presented in Table 1.

**Table 1:** Binomial analysis of pre-consultation expectations

Pre-consultation expectation	Expected [*N* (%)]	Not expected [*N* (%)]	Binomial test (*p* value)
History taking	61 (85.9%)	*n*=10 (14.1%)	*P*<0.001
Clinical examination	65 (91.2%)	*n*=6 (8.5%)	*P*<0.001
Medical investigations	59 (83.1%)	*n*=12 (16.9%)	*P*<0.001
Diagnose problem	52 (73.2%)	*n*=19 (26.8%)	*P*<0.001
Prescribe medication	62 (88.6%)	*n*=8 (11.4%)	*P*<0.001
Case to be discussed with doctor	37 (52.9%)	*n*=33 (47.1%)	*P*=0.720
Onward referral	59 (83.1%)	*n*=12 (17.6%)	*P*<0.001

Table 1 shows a significant (*P*<0.001) majority of respondents expected the nurse practitioners to engage in advanced clinical practice activities. Only the pre-consultation expectation for a case to be discussed with a doctor by the nurse practitioner did not have a significant higher proportion of patients expecting this activity in their consultation (*P*=0.720). For this particular expectation, there was an almost even split (Yes 52.9%/No 47.1%) amongst the respondents as to whether they thought the nurse practitioner would discuss their case or that of the person they were accompanying with a doctor. This result suggests that many respondents were not fully conversant with the independent, autonomous nature of the nurse practitioner role, despite most of them clearly expecting the nurse practitioner to engage in areas of advanced clinical practice such as clinical examination, diagnosis, and prescribing, as can be seen in the preceding expectations responses. All respondents either agreed (*n*=20, 30.3%) or strongly agreed (*n*=46, 69.7%) that their overall expectations of coming to see the nurse practitioner had been met.

### Post-consultation satisfaction

The descriptive statistics for the satisfaction scores are displayed in Table 2. These satisfaction mean scores indicated that both general satisfaction and communication satisfaction scores were high.

**Table 2:** Descriptive statistics for general satisfaction and communication satisfaction scores

Descriptive statistics	General satisfaction score (maximum possible 85)	Communication satisfaction score (maximum possible 30)
Mean (SD)	78.48 (6.68)	26.37 (2.75)
Median (quartiles)	81.0 (73.0, 84.0)	27.0 (24.0, 28.5)
Skewness statistic	−1.024	−0.636

No significant differences in satisfaction scores were attributed to demographics, participants consulting with different nurse practitioners, or being video recorded or not being video recorded. The respondents’ satisfaction scores were also compared with their pre-consultation expectations for the nurse practitioners utilising advanced clinical practice skills and for respondents’ expectations for the nurse practitioners to discuss their case with a doctor. The only pre-consultation expectation with a significant difference was for general satisfaction in relation to diagnosis expectations; the median general satisfaction score was significantly higher (*P*=0.043) for those with diagnosis expectations (median 82.0) than the median score for those not expecting the nurse practitioner to diagnose their problem (median 75.0). From this analysis, there is no evidence to suggest that those patients with lower expectations are more satisfied than patients with higher expectations. Hence it appears that the high levels of satisfaction with nurse practitioner consultations cannot simply be explained by patients having low expectations of nurse practitioner consultations that have been exceeded.

### Patient enablement

The descriptive statistics for the enablement score are displayed in Table 3. No significant variations in enablement scores were noted in relation to being video recorded, demographics, or nurse practitioner consulted with. In relation to consultation expectations and enablement, there were no significant differences in respondents’ expectations for the occurrence of advanced practice activities in their consultations and their reported post-consultation enablement. So in this study patients’ pre-consultation expectations do not appear to affect their subsequent evaluations of post-consultation enablement.

**Table 3:** Descriptive statistics for patient enablement scores

Descriptive statistics	Enablement score (maximum possible 12)
Mean (SD)	6.08 (3.40)
Median (quartiles)	6.0 (4.0, 8.0)
Skewness statistic	0.066

A correlational analysis of the satisfaction and enablement scores was performed, using Spearman’s *ρ* correlation coefficient, to ascertain if any associative relationship existed between the two outcome variables. This analysis showed a significant, small to moderate, positive correlation of 0.427 (*P*=0.005) between general satisfaction and enablement, and a non-significant, small, positive correlation of 0.216 (*P*=0.150) between communication satisfaction and enablement; so the more generally satisfied a patient is they correspondingly feel more enabled.

The mean video recorded consultation time length was 10.97 min (SD 4.13). The consultation time lengths were correlated, using Spearman’s *ρ* correlation, with the scores for general satisfaction, communication satisfaction, and enablement, to see if there was any relationship between consultation time lengths and those outcomes variables. A non-significant, small, positive correlation of 0.209 (*P*=0.326) for general satisfaction and consultation time length was noted. For communication satisfaction and consultation time length, there was a very small, non-significant slightly positive correlation of 0.014 (*P*=0.946). Both of these correlations of consultation time lengths and satisfaction scores indicate that in this study there is no significant association between consultation time lengths and post-consultation satisfaction scores. These findings do not support the notion that longer consultation times are significantly associated with increased patient satisfaction.

There was a non-significant, small negative correlation for enablement and consultation time length of −0.104 (*P*=0.644). This correlational finding indicates that in this study longer consultation times did not significantly increase enablement.

## Discussion

In this current study on exploring the relationship between pre-consultation expectations and post-consultation satisfaction, the finding that increased satisfaction is generally reported when patients expect the nurse practitioner to use advanced practice skills, does not provide support for Redsell *et al.*’s (2007b) previously discussed assertion that patients’ lowered probability expectations of nurses’ abilities in consultations may lead to increased satisfaction. Indeed it seems in this study that the opposite effect has been found; people who are actually expecting their nurse practitioner to utilise advanced clinical practice skills are generally more satisfied when their expectations are met, than those people who are not actually expecting the nurse practitioner to utilise advanced clinical practice skills. Similarly, in relation to pre-consultation expectations and post-consultation enablement where respondents actually expected the nurse practitioners to demonstrate advanced practice care, post-consultation enablement was mainly reported as being higher. These findings, coming a decade after Redsell *et al*.’s (2007b) study may potentially arise from the public’s increased awareness of nurses working in advanced clinical roles, and also because the clinic where the current study was conducted was a nurse practitioner-led general practice clinic, where most patients usually consult with nurse practitioners rather than general practitioners.

The mean consultation time length noted in this study for the video recorded consultations was 10.97 min. No significant correlation was found between increased consultation time lengths and post-consultation patient satisfaction and enablement scores. This finding is in contention to the findings of previous studies of nurse practitioner consultations that increased consultation time lengths for nurse practitioners are associated with high levels of patient satisfaction (Kinnersley *et al.*, 2000; Laurant *et al*., 2005; Seale *et al*., 2005; 2006). How does this study’s mean consultation time length of 10.97 min compared with the average length of GP consultations? NHS England has reported that the mean consultation time length for GPs is ~12 min (Parkinson, 2013). The mean consultation time of 10.97 min noted for the nurse practitioners in this study compares very favourably with the similar mean GP consultation time length quoted by NHS England, with a one-sample *t*-test showing this study’s consultation time length is not significantly different to the time length of 12 min quoted by NHS England (*P*=0.280) (95% confidence interval 9.27, 12.82).

The current study’s mean patient enablement score of 6.08 is 1.48 points higher than the combined mean enablement score (4.6) of previous PEI studies (Venning *et al*., 2000; Simmons and Winefield, 2002; Denley *et al*., 2003; Ford *et al*., 2003; MacPherson *et al*., 2003; McKinley, 2004; Price *et al*., 2006; Haughney *et al*., 2007; Wensing *et al*., 2007; Adžic *et al*., 2008; Pawlikowska *et al*., 2009; Hudon *et al*., 2011; Mercer *et al*., 2012; Pawlikowska *et al*., 2012; Brusse and Yen, 2013; Rööst *et al*., 2015). A one-sample *t*-test shows this study’s mean enablement score is significantly higher (*P*=0.003) than 4.6 (the combined mean of previous studies), and hence indicates the participants of this study did feel more highly enabled after consulting with a nurse practitioner than other participants did after consulting with other types of clinicians in previous studies of patient enablement (95% confidence interval 5.12, 7.03). In comparison to studies of patient enablement after seeing a GP, there are far fewer available quantitative studies of patient enablement after consulting with a nurse practitioner (Frost *et al*., 2015; Frost *et al*., 2017). Venning *et al*.’s (2000) comparative RCT of nurse practitioners did assess patient enablement using the PEI and found that 335 patients consulting with a nurse practitioner had a mean enablement score of 4.92. Using a one-sample *t*-test it can be seen that this current study, albeit with a smaller sample size of 51 patients, had a mean level of enablement score of 6.08 that was significantly higher (*P*=0.019) than the mean enablement score after seeing a nurse practitioner that was reported in Venning *et al*.’s (2000) study. Venning *et al*. (2000) only sampled same day consultations, whereas this study included both same day consultations and pre-booked appointments. Furthermore, the nurse practitioners in Venning *et al*.’s (2000) study had to get their prescriptions authorised by doctors as full-formulary access independent nurse prescribing did not exist in the United Kingdom until May 2006 (Courtenay and Carey, 2007). Contrastingly the nurse practitioners in this study were able to make fully autonomous diagnostic and prescribing decisions for patients with both acute and long-term conditions, which may have had a differential impact on patients’ evaluations of post-consultation enablement.

In this study correlational analysis was used to explore the relationship between patient enablement and patient satisfaction, to investigate if any relationship exists between enablement and satisfaction. This correlational analysis found general satisfaction was significantly positively correlated with enablement, and also a non-significant small-moderate positive correlation between communication satisfaction and enablement. These findings indicate the more enabled a patient feels, the more satisfied they also feel. However, these findings, being based solely on correlational analyses, do not provide causative evidence that high enablement causes high satisfaction or vice versa. Studies of patient enablement have found that patients being previously familiar with their consulting clinician predict higher enablement (Howie *et al*., 1998; Brusse and Yen, 2013). Correspondingly in this study, many of the participants knew the nurse practitioners they were consulting with, as the research setting was their registered general practice clinic where they attended for repeat visits. Furthermore, a primary care-based survey study of predictors of patient satisfaction has found that the presence of unmet expectations post-consultation is a significant predictor of patient dissatisfaction (Jackson *et al*., 2001). In the current study, 100% of respondents felt their expectations of coming to see the nurse practitioner had been met, which in turn may have contributed to the study’s reported high levels of satisfaction. It can, therefore, be speculatively postulated that this study’s observed the effect of enablement and satisfaction scores increasing with one another seen can be explained by the combination of registered patients’ familiarity with the nurse practitioners, and a lack of unmet expectations amongst those patients.

## Limitations

The sample size for the survey part of the case study was relatively small, at only 71 completed questionnaires. The modest ambition of 100 completed questionnaires was not achieved. The small sample size was dictated by the practicalities of a single researcher conducting the study in just one primary care clinic. However, this small sample size does raise concerns about the power of the study and the consequent need for caution in the interpretation of statistical tests. Some of the analyses completed using the questionnaire data were based on the smaller sub-sample of 26 video recorded questionnaire respondents, such as when patient satisfaction and enablement scores were compared against the time lengths of the video recorded consultations. Compared with other studies measuring patient satisfaction and enablement the sample numbers used in this study are relatively small, as for example, Agosta’s (2009b) patient satisfaction survey had 300 respondents, and the majority, though not all, previous surveys of patient enablement had samples of either hundreds (Wensing *et al*., 2007) or thousands of patients (Mercer *et al*., (2012). However, in contrast to the overall scope of this case study, none of these larger studies have attempted to link satisfaction and enablement to the detailed process content of consultations which requires observation and detailed frequency occurrence analysis of interactions and would be very difficult to achieve on a large scale beyond the 30 videoed nurse practitioner consultations sampled in the overall case study this survey component being reported forms part of (Barratt, 2016).

## Conclusion

In relation to patient satisfaction and patient enablement, this case study-based survey has found high levels of patient satisfaction and enablement after consulting with nurse practitioners. It would be beneficial to repeat the survey used in this case study with a larger, more varied sample of respondents who see nurse practitioners for general practice care, so that the findings of this study in relation to high satisfaction and enablement scores, and comparisons with consultation time lengths, can either be further supported or modified. The replication of the survey on a larger scale would also be particularly useful, first to further examining whether other patients do not fully understand the autonomous nature of the nurse practitioner role as is elicited in the pre-consultation expectations section of the questionnaire, and second to determine whether a significant positive association still exists between patient enablement and satisfaction.

In this study it has been shown accurate patient expectations of nurse practitioner consultations boost patient satisfaction and enablement, so public education strategies to promote awareness of the discrete nature of the nurse practitioner role should be implemented, as a plethora of role titles exist for describing nurses working at an advanced level of practice, which taken together with uncertain expectations of the nurse practitioner role can confuse patients (Leary *et al*., 2017). From a clinical practice perspective, the findings suggest that increased satisfaction and enablement can be engendered in patients by nurse practitioners independent of consultation time length durations, and indeed can even be achieved with shorter consultation time lengths. Accordingly, the processes of how consultations are conducted may be more important than their time lengths for optimising patient satisfaction and enablement. Furthermore, the findings of this study endorse workforce development strategies across secondary and primary health care for deploying nurse practitioners (Kilpatrick *et al*., 2011; Sangster-Gormley *et al*., 2015; Hill, 2017), as they can evidently optimise consultation outcomes such as patient satisfaction and enablement without the resource implications of longer consultation times.
